# Investigations on the Performance of a Downhole Electric Heater with Different Parameters Used in Oil Shale In Situ Conversion

**DOI:** 10.1021/acsomega.4c03425

**Published:** 2024-08-08

**Authors:** Dazhong Ren, Zhendong Wang, Fu Yang, Hao Zeng, Zengzeng Zhang

**Affiliations:** † State Key Laboratory of Shale Oil and Gas Enrichment Mechanisms and Effective Development, Beijing 100083, China; ‡ State Center for Research and Development of Oil Shale Exploitation, Beijing 100083, China; § Shaanxi Key Laboratory of Advanced Stimulation Technology for Oil & Gas Reservoirs, Xi’an Shiyou University, Xi’an 710065, China; ∥ Key Laboratory of Thermo-Fluid Science and Engineering, Ministry of Education, School of Energy and Power Engineering, Xi’an Jiaotong University, Xi’an 710049, China; ⊥ School of Civil Engineering and Geomatics, Shandong University of Technology, Zibo 255049, China

## Abstract

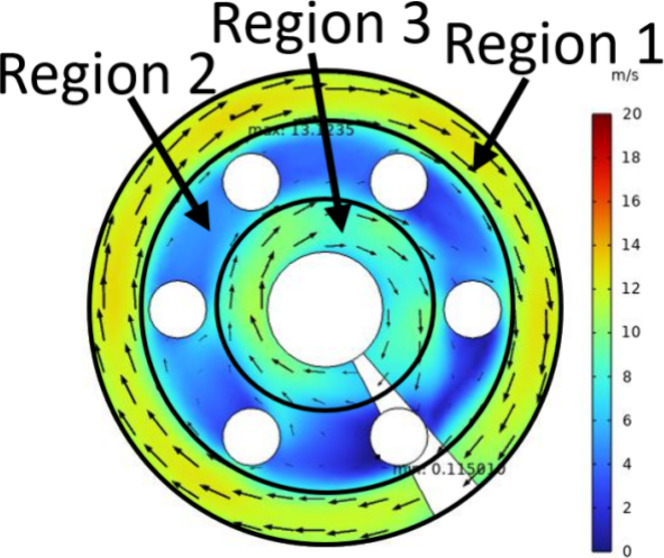

In situ conversion is the most potential technology for efficient and clean development of oil shale, and a downhole electric heater is key equipment for clean, efficient, and low-carbon in situ conversion. Three electric heating rods with different diameters are used to explore their influence on heater performances. The simulation results indicate that increasing the diameter of the heating rod helps to increase the minimum and maximum velocity of shell-side air, and the maximum velocity of H110-24 is 16.34 m/s, which is 1.25 and 1.13 times those of H110-16 and H110-20, respectively. In addition, the location of the local high temperature zone coincides with the area with low air flow velocity, and increasing the diameter of the heating rod can effectively reduce the heating rod surface temperature during high-power heating. Moreover, at the same heat flux, the heat transfer coefficients of H110-24 and H110-20 are 44.82–48.49% and 87.52–95.48% higher than those of H110-16, respectively. With the same heating power, the heat transfer coefficients of heaters have the same trend, indicating that the heat transfer coefficient of the heating rod can be effectively improved by increasing the diameter of the heating rod. Finally, the newly defined comprehensive performance is used to evaluate the heaters with different heating parameters. Increasing the heating power can improve the comprehensive performance of the heater, but the most effective way is to increase the diameter of the heating rod. With the same heating power, the new comprehensive performance of H110-24 and H110-20 is 48.38–52.34% and 87.29–95.19% higher than that of H110-16, respectively, and the electric heating rod with the diameter of 20 mm has the best performance.

## 1 Introduction

With the rapid development of human society, global energy consumption has sharply increased. It is estimated that global energy consumption will increase by 34% in the next two decades.^1^ In recent years, the proportion of easily exploitable light crude oil in crude oil consumption has gradually decreased, and unconventional reservoir extraction technology has received increasing attention.^2,3^ Unlike light crude oil, unconventional reservoirs (such as heavy oil and oil shale) require heat treatment to reduce their viscosity or convert kerogen into petroleum.^4,5^ Generating heat carriers using downhole heaters is an efficient and economical method for heating unconventional oil reservoirs.^6^

Traditional heavy oil development usually uses surface heaters to inject high-temperature steam underground.^7^ According to the different fuels, surface heaters can be divided into coal-fired boilers, oil-fired boilers, and gas boilers. The boilers include four parts: the radiation section, convection section, combustion system, and control system.^8^ The radiation section is generally composed of a cylinder, a tube bundle, and an inner insulation layer. This section mainly heats steam through thermal radiation. The convection section is mainly composed of light tubes or finned tubes, which mainly generate steam or high-temperature gas through thermal convection.^9^ The steam dryness provided by coal-fired boilers is generally greater than 80%. The surface heater technology is relatively mature, but it is prone to dust accumulation in the heat exchange section and has a high maintenance frequency.^10^ The high-temperature fluid generated by the surface heater needs to be transported to the target formation through a heat injection pipeline, and the high-temperature fluid will generate a large amount of heat loss during the transportation process. Therefore, the surface heater is suitable for shallow burial depth or lower heating temperature required in the formation (<350 °C).

According to the energy source of downhole heaters, they can be divided into downhole combustion heaters and electric heaters. Xu and Chen^11^ developed a downhole steam generator, which is a combination of an efficient combustion chamber and a gas water heat exchanger. The high-temperature flue gas and steam are mixed to form high-temperature steam through the annular gap water cooling combustion chamber. This type of downhole steam generator has undergone only laboratory tests under normal pressure conditions, and no further reports on tests under high pressure and on-site tests have been found. Schicks^12^ developed a counter-current heat exchange reactor that utilizes in situ combustion of CH_4_ to thermally stimulate deposits containing hydrates. They found that Pt/Ir loaded on ZrO_2_ operated stably at 450 °C and exhibited the highest CH_4_ conversion rate (>99%). Oil shale is a type of low-permeability and low-thermal-conductivity reservoir. After hydraulic fracturing, high-temperature fluids require higher pressure to heat oil shale through thermal convection. If a high-temperature fluid forms a thermal short circuit in the oil shale layer, the combustion reaction will quickly stop, and secondary ignition is difficult, which affects the stability of the downhole in situ conversion process.^13,14^ Therefore, the downhole combustion heaters are not suitable for in situ conversion of unconventional reservoirs.

To solve the problems of downhole combustion heaters, researchers have proposed downhole electric heating heaters. Conductive downhole electric heating is a method of heating the formation by inserting an electric heating rod into the formation through thermal conduction. Hao et al.^15^ designed bare electrode heaters with U-shaped tubes and vacuum heating tubes and simulated the performance of the heaters using MATLAB. The effects of the properties of the heater material and the type of electric heating rod on the temperature distribution of the heating electrodes are studied. Bybee^16^ studied the effect of heating parameters of wellbore heaters on heavy oil production through numerical simulation. Zhu et al.^17^ studied the effects of electrode arrangement and input voltage on oil recovery. They found that in the initial stage, it can lead to a rapid oil response rate, and the oil recovery rate increases with the increase of input voltage. Hassanzadeh and Harding^18^ studied the heat conduction process during in situ electric heating of oil sands. They found that the electric heater (electrode) reached high values in the loose formations and high gas saturation. However, due to the small heat transfer area of bare electrodes, the heat transfer efficiency of heat conduction is low, and heating unconventional reservoirs with electrodes always consumes a lot of time. In addition, bare electrodes without enhanced heat transfer structures are prone to thermal damage under high heat flux, which is caused by local hot spots. Therefore, bare electrode heaters are not suitable for generating large amounts of high-temperature fluids over a short period of time.

To address the aforementioned issues, researchers have introduced enhanced heat transfer structures. In addition, the flow pattern of the fluid on the shell side of the downhole electric heater is similar to that of the shell and tube heat exchanger (STHX), so the advanced heat transfer enhancement technology applied in the heat exchanger can be applied to the downhole electric heater. Chen et al.^19^ conducted experiments to explore the thermal performance of a trisection spiral baffle heat exchanger. They found that the comprehensive performance decreases with the increase of the mass flow rate and baffle incline angle, and the comprehensive performance of heat exchangers with a helix angle of 12° is about 50% higher than that of segmental heat exchangers. Duan et al.^20^ studied the thermal performance of noncontinuous spiral baffle heat exchangers with different helix angles, a continuous connection method, and an intermediate overlap method. According to their work, a heat exchanger with a helix angle of 40° has the highest comprehensive performance. In addition, the continuous connection method has a lower local resistance and pressure drop, and its performance is better than the intermediate overlap method. Du et al.^21^ simulated continuous helical baffle heat exchangers with different elliptical tube arrangements. They claim that the angle of the elliptical tube arrangement has a significant impact on the performance of the heat exchanger. Guo et al.^4,14^ studied downhole electric heaters with continuous helical baffles and explored the effect of the packer position on the performance of downhole heaters. They pointed out that a downhole electric heater with a helical pitch of 50 mm and a packer installed at its outlet is the best solution to achieve optimal comprehensive performance and lower total cost. With the help of different enhanced heat transfer structures, many studies have effectively improved the heat transfer capacity of heat exchangers or electric heaters and reduced the surface temperature of heat transfer tubes or electric heating tubes. However, in current research, there is still a very high temperature at the end of the electric heating rod. In practical applications, the temperature of electric heating rods not only affects the manufacturing cost and lifespan of heaters but also affects their ability to be widely applied in the field of in situ conversion.

The parameters of the electric heating rod and the enhanced heat transfer structure are two key structural parameters that influence the surface temperature of the electric heating rod. In our previous research, we conducted a detailed study on the enhanced heat transfer structure. Therefore, this paper mainly studies the influence of different heating rod diameters under different heating parameters and studies the shell-side flow field, temperature field, electric heating rod surface temperature, and traditional comprehensive performance in detail. Finally, a new comprehensive performance index is proposed to evaluate the performance of the heater studied, and the optimum diameter of the heating rod is obtained under the experimental conditions.

## 2 Modeling and Numerical Methods

### 2.1 Physical Models

**Figure 1:**
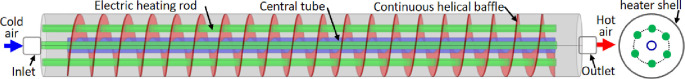
Structure diagram of the downhole electric heater.

In this paper, downhole electric heaters with different electric heating rod diameters are numerically simulated. For the downhole electric heater, the continuous helical baffle deflects more in the center of the spiral, and the air flow rate is low, so the center tube is set here. The structure diagram of the downhole electric heater is shown in Figure 1.

The geometric model is established by the inventor, and the shell-side flow and heat transfer of the heater with different parameters are simulated by COMSOL Multiphysics. Table 1 illustrates the downhole electric heater with different parameters.

**Table 1:** Downhole Electric Heater with Different Parameters^a^

	dimensions and description		
item	H110-16	H110-20	H110-24
shell inside diameter (mm)	131	131	131
shell outside diameter (mm)	139	139	139
inlet and outlet nozzle diameter (mm)	25.4	25.4	25.4
shell length (mm)	1100	1100	1100
helical baffle length (mm)	1000	1000	1000
helical baffle thickness (mm)	2	2	2
baffle pitch or helical pitch (mm)	110	160	210
central tube outside diameter (mm)	32	32	32
heating rod length (mm)	1050	1050	1050
effective length of the heating rod (mm)	1000	1000	1000
heating rod outside diameter (mm)	16	20	24
number of heating rods	6	6	6
heating rod layout pattern	60° rotation in a single row		

a H110-20-1 represents the H110-20 with the same heat flux of H110-16, and H110-20-2 represents the H110-20 with the same heating power of H110-16.

### 2.2 Simulation Methods

The coupled method is used to determine the heat transfer process between the electric heating rods and shell-side air. Additionally, the SIMPLE algorithm is used to address the coupling of the velocity and pressure, while the second-order upwind scheme is applied to solve the momentum, energy, and turbulence parameters.^22^ Furthermore, in the continuity equation, the residual values of *u*_*i*_, *k*, and ε are set on the order of 10^–5^, and the energy residual value is set on the order of 10^–6^.

In the process of numerical calculation, when all parameters reach the corresponding residual value, the calculated result is judged to converge. The numerical analysis of this study is based on four assumptions: (1) the shell-side air is regarded as fully developed turbulence and is in a stable state; (2) the shell-side air is regarded as incompressible; (3) the heat dissipation of the surface of the heater shell is ignored; (4) the electric heating rod is regarded as a wall with a constant heat flux.^23^

Because the flow pattern of heater shell-side air is spiral flow, the renormalization group (RNG) *k* – ε model is used in the simulation process. In addition, the options “enhanced wall treatment” and “thermal effects” are selected.^24^ The universal governing equation of the mass, energy, momentum, and RNG *k* – ε turbulent viscosity is expressed as follows^25^:
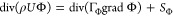
1where *U* is the velocity vector; Φ is a universal variable representing *u*_*i*_, *T*, *k*, and ε or another variable; Γ_Φ_ is a generalized diffusion coefficient; and *S*_Φ_ is a generalized source term.

The heat transfer coefficient and pressure drop are calculated by eqs 2 and 3.^26,27^
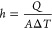
2
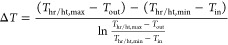
3
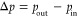
4

Equation 5 shows the traditional comprehensive performance of the heater; in the performance studies of heat exchangers, the heat transfer coefficient and pressure drop are the key factors.^28,29^ However, in the oil shale in situ conversion process, we are more concerned about the heat transfer coefficient and the surface temperature of the electric heating rod. Therefore, the ratio of the heat transfer coefficient and the average surface temperature of the electric heating rod is used as a new comprehensive performance index in eq 6.
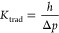
5
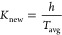
6

### 2.3 Simulation Validation

**Figure 2:**
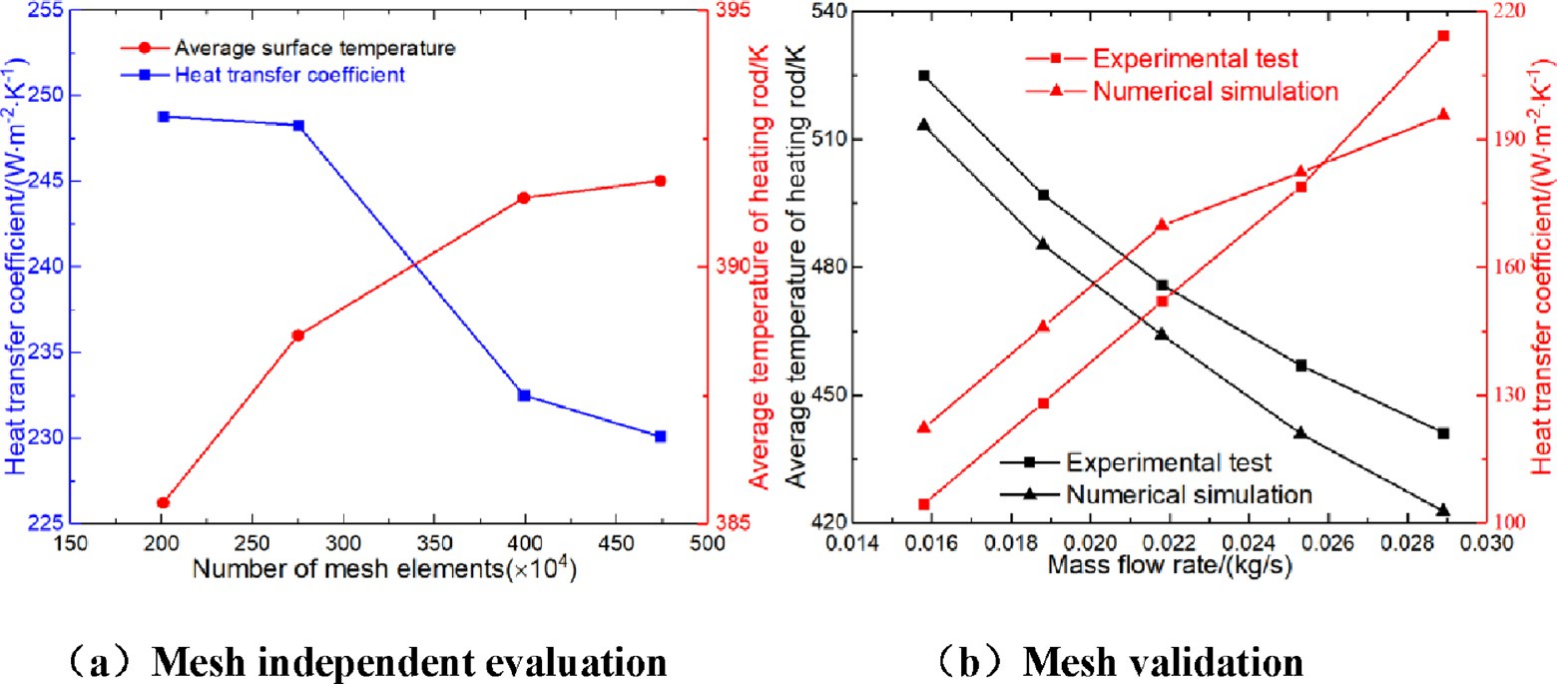
Mesh independent evaluation and validation (a,b).

In the calculation domain of the heater shell side, the central tube, the heater shell, and the surface of the heating rod are regarded as nonpenetration, nonslip, and adiabatic boundary conditions, and the surface of the heating rod and the continuous helical baffle are regarded as nonpenetration, nonslip, and coupled heat transfer boundary conditions. In addition, the numerical simulation uses an unstructured tetrahedral mesh for mesh partitioning and mesh refinement near the wall. Before calculating the flow and heat transfer processes of the heater, the mesh independent evaluation is carried out to obtain the optimal mesh parameters. Under the condition of a mass flow of 0.0382 kg/s and a heat flux of 16,578.64 W/m^2^, four models with different mesh quantities are calculated, and the results are shown in Figure 2. It is obvious that with the increase of the number of grid cells, the heat transfer coefficient of the heater and the average surface temperature change rate are 0.21, 6.35, and 1.04% and 0.85, 0.69, and 0.08%, respectively. The heat transfer coefficient and the average surface temperature difference of the last two grids are less than 2.0%. Considering the calculation efficiency and accuracy, a grid number of 4.74 M is more appropriate. By comparing the numerical simulation with the experimental test,^13^ we found that the maximum deviation of the heat transfer coefficient is 14.6%, and the average deviation is 9.6%. The maximum deviation of the average temperature is 4.2%, and the average deviation is 3.0%. Considering that the deviation is within an acceptable range, the current mathematical modeling method is reliable.

## 3 Results and Discussion

### 3.1 Flow Fields

**Figure 3:**
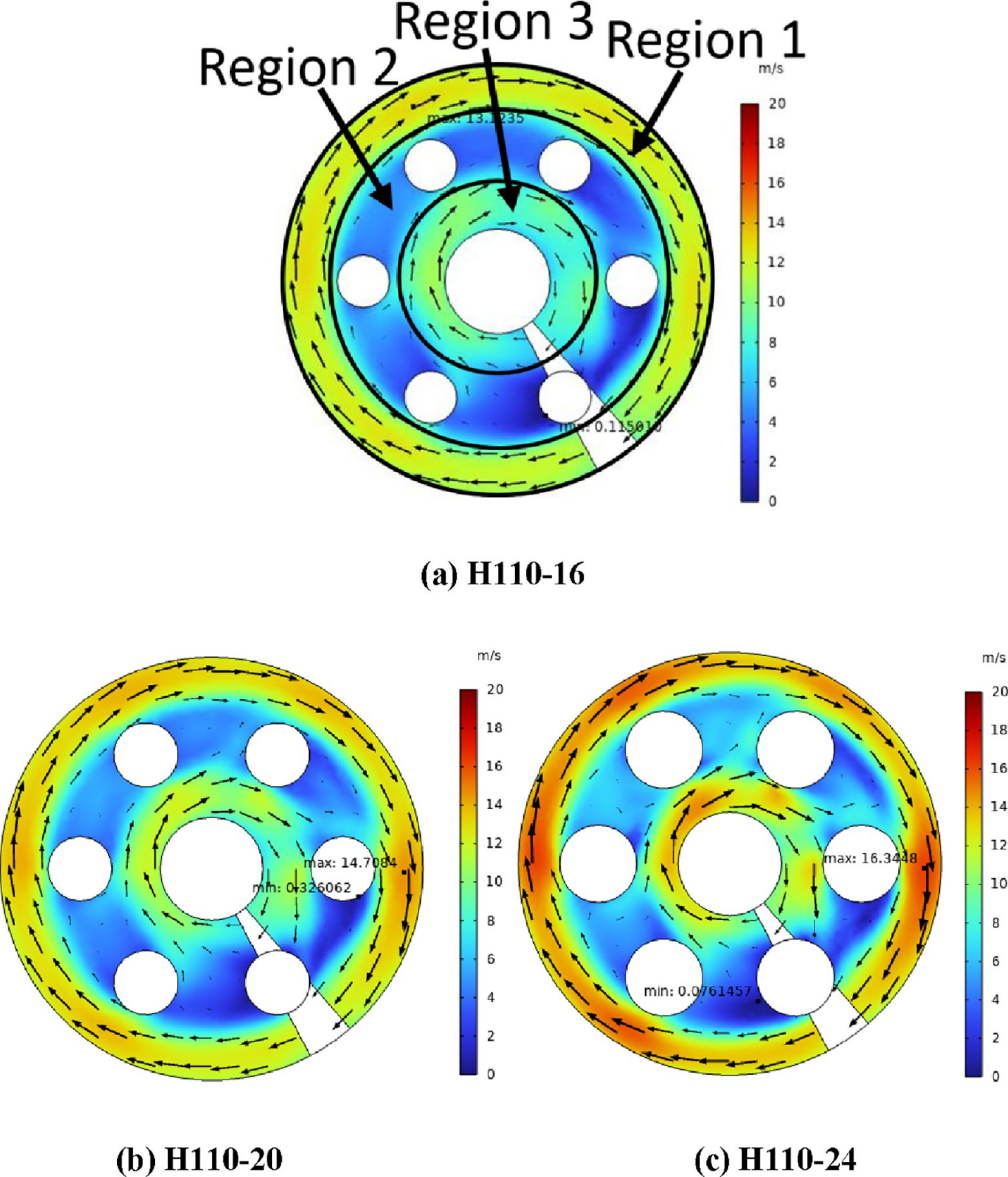
(a–c) Air flow velocity of the heater with the same heating power (*Z* = 260 mm).

Figure 3 shows the air flow velocity at the cross section of different heaters (*Z* = 260 mm) with the heating power. In Figure 3, the air scours the heating rod at an uneven speed, and the velocity can be divided into three regions. In Figure 3a, region 1 has the highest air velocity (the maximum value is 28.01 m/s), while region 2 has the lowest. In region 2, the air flow velocity is the largest at the edge of the windward side of the electric heating rod, and the smallest at the leeward side (the minimum value is 0.12 m/s). When there is no heating rod in the shell side, the flow pattern of air is a standard spiral flow; under the action of a centrifugal force, the air velocity in region 1 is the highest, and that in region 3 is the lowest. The cross section of the electric heating rod is circular, and there is a flow dead zone on the leeward side, so the air velocity in region 2 is the lowest.

As shown in Figure 3, with the increase of the electric heating rod diameter, the effective flow area of the air decreases, and the nonuniformity of the air velocity increases gradually. The maximum velocity of region 1 increases with the increase of the heating rod diameter, and the increasing range is gradually larger. Meanwhile, with the increase of the heating rod diameter, the consistency of the velocity of regions 3 and 1 is enhanced. With the increase of the heating rod diameter, the minimum flow velocity in region 2 increases and then decreases. The maximum velocity of H110-24 is 16.34 m/s, which is 1.25 and 1.13 times those of H110-16 and H110-20, respectively.

**Figure 4:**
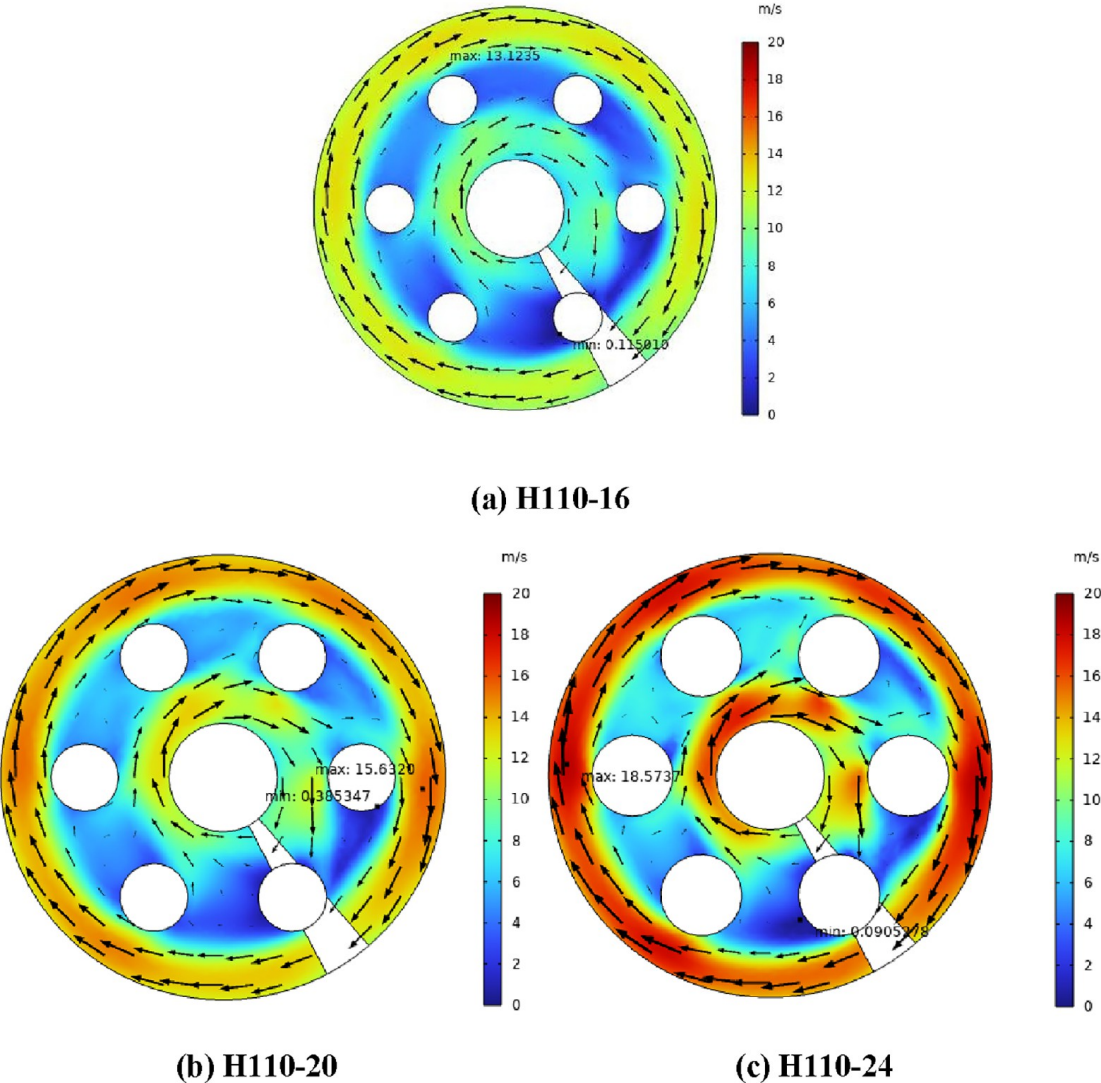
(a–c) Air flow velocity of heater with the same heat flux (*Z* = 260 mm).

Figure 4 shows the air flow velocity at the cross section of different heaters (*Z* = 260 mm) with the heat flux of 16,579 W/m^2^. Compared with H110-16, the maximum air velocities of H110-20 and H110-24 increased by 18.81 and 41.5%, respectively. Compared with Figure 3, with the increase of heat flux, the maximum air velocity of H110-20 and H110-24 increases by 6.25 and 13.65%, respectively. Under the same heat flux condition, the larger the diameter of the electric heating rod, the higher the heating power, the higher the shell-side air temperature, the lower the density, and the higher the air velocity. At the same time, the diameter of the electric heating rod increases, the effective air flow area decreases, and the air velocity further increases. Therefore, the diameter of the electric heating rod and heat flux have a significant influence on the maximum velocity of shell-side air.

**Figure 5:**
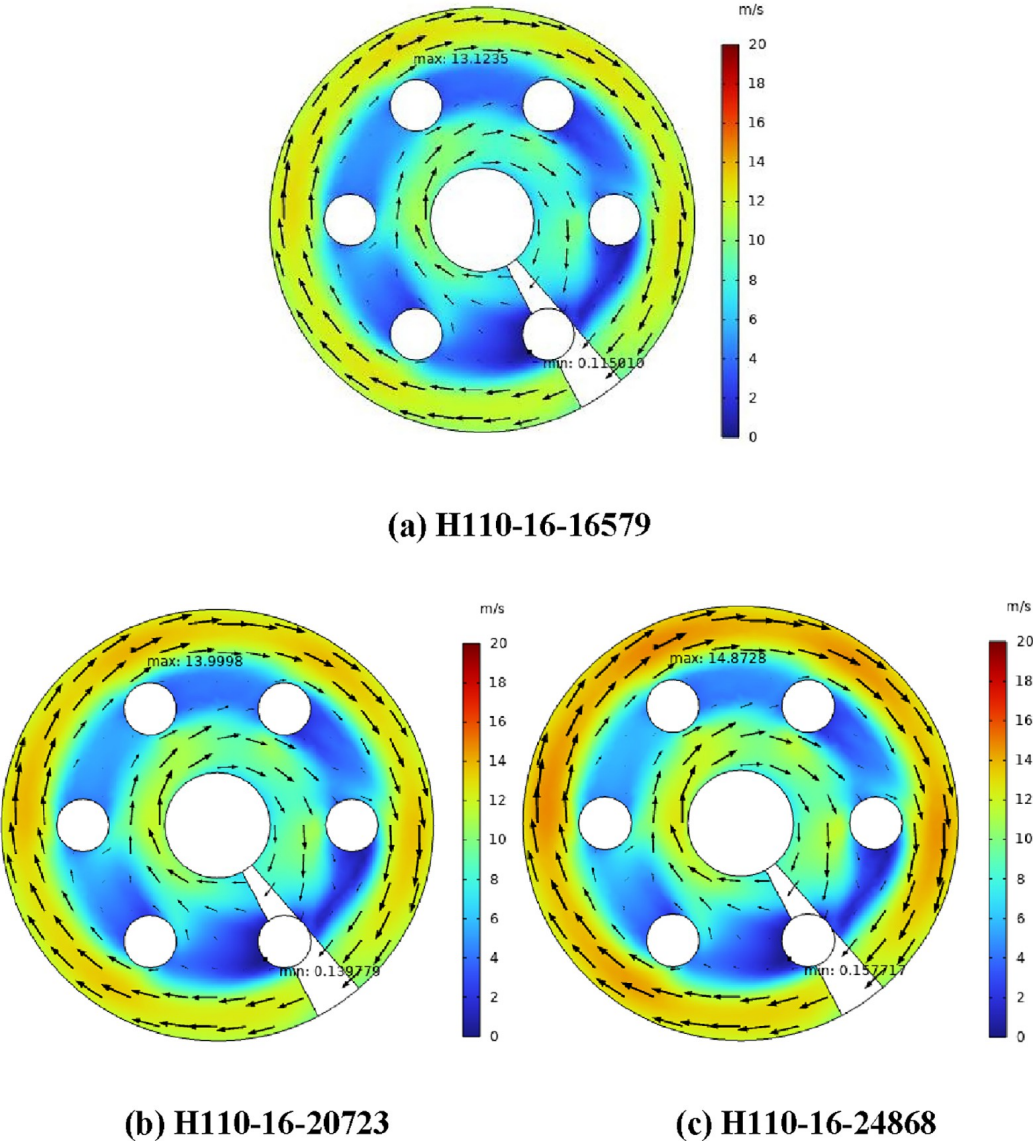
(a–c) Air flow velocity of the heater with the same heat flux (*Z* = 260 mm).

H110-16 are set with three different heat flux to further study the influence of heating power on the maximum air velocity. In Figure 5, the heating power of H110-16-20723 and H110-16-24868 is the same as that of H110-20 and H110-24 in Figure 4, respectively. H110-16-20723 represents the electric heating rod of H110-16 that heats the shell-side air with the heat flux of 20,723 W/m^2^. Compared with H110-16-16579, the maximum air velocity of H110-16-20723 and H110-16-24868 increased by 6.68 and 13.33%, respectively, which is consistent with the changes of H110-20 and H110-24 in Figure 4, indicating that increasing the heating power alone cannot effectively increase the maximum air velocity. In addition, the larger the diameter of the heating rod, the greater the increase in the maximum air velocity, which indicates that increasing the diameter of the electric tube is an effective way to increase the maximum air velocity.

### 3.2 Temperature Fields

**Figure 6:**
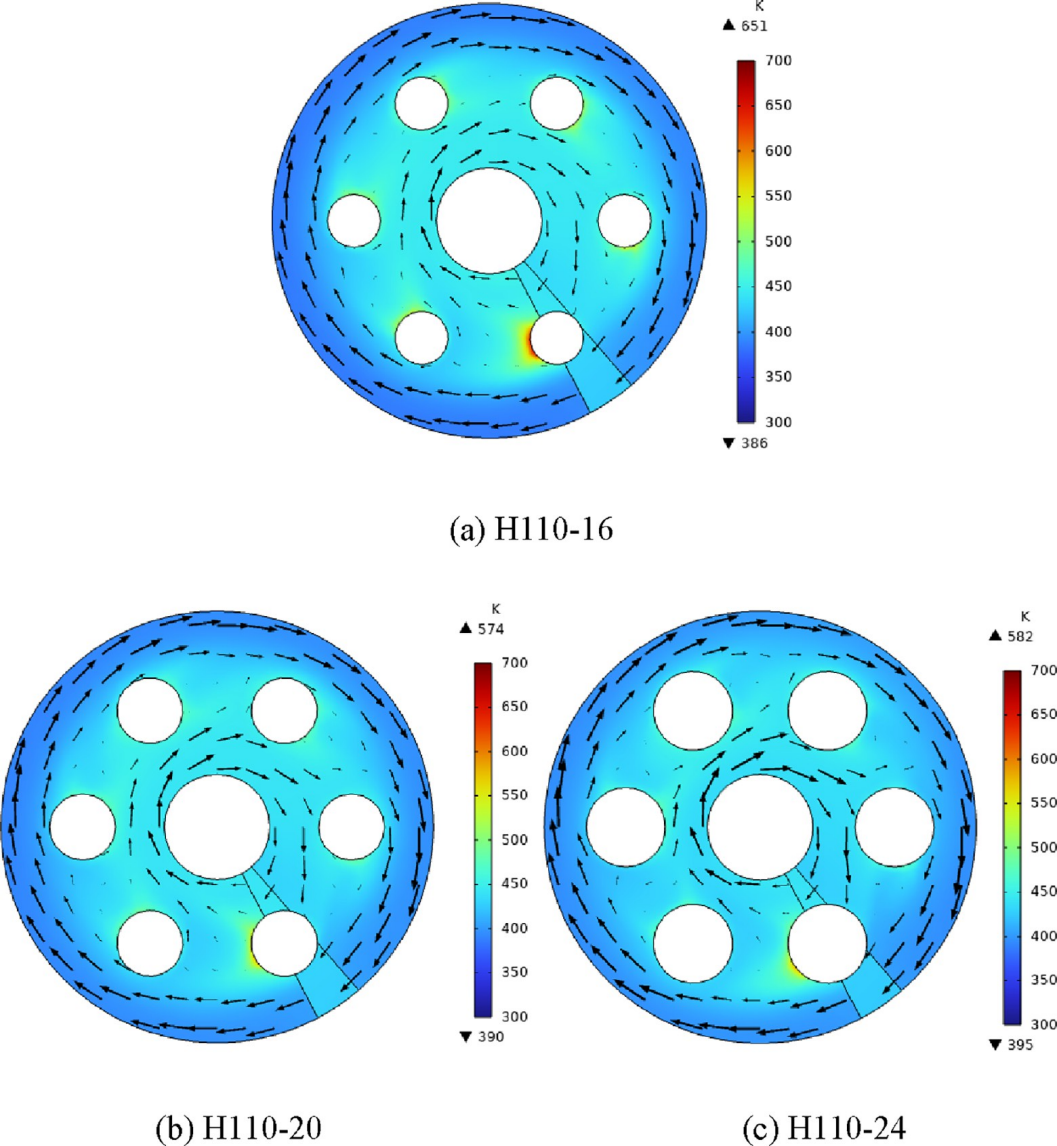
(a–c) Temperature superposed on air velocity nephograms (*Z* = 260 mm).

Figure 6 presents the temperature superposed on air velocity nephograms of different heaters with the same heating power. It can be found that the local high temperature zone exists on the leeward side of the electric heating rod. Compared with Figure 3, it can be seen that the location of the local high temperature zone coincides with the area with low air flow velocity, and the low air flow velocity weakens the ability of air to carry out heat exchange. Therefore, the local high temperature zone of the electric heating rod exists on the leeward side of the heating rod. With the increase of the heating rod diameter, the minimum flow velocity on the leeward side is lower, so the temperature in the local high temperature region is higher. With the increase in the heating rod diameter, the maximum temperature of the section decreases first and then increases, and the minimum temperature increases slowly.

**Figure 7:**
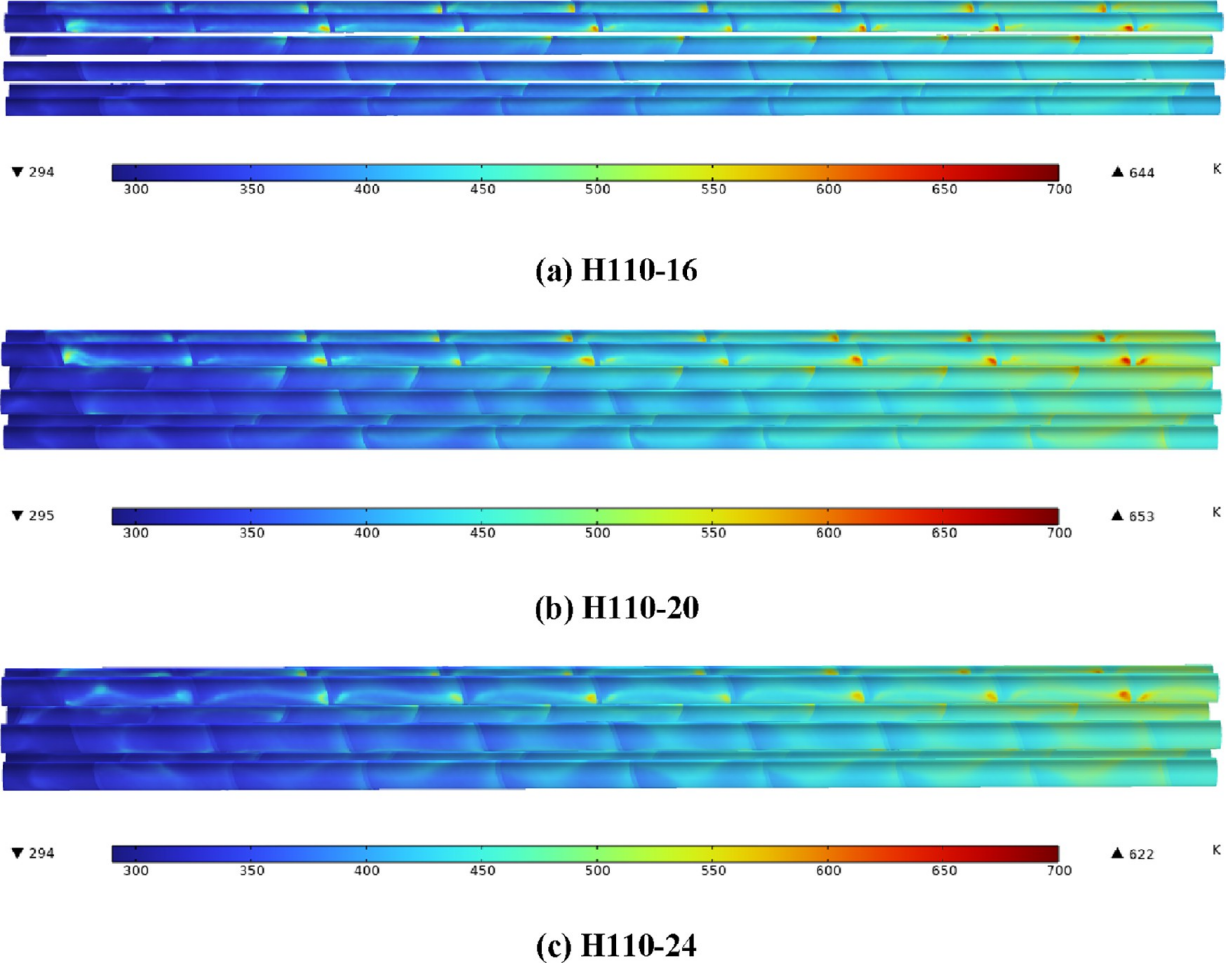
(a–c) Electric heating rod surface temperature nephograms.

Figure 7 shows the surface temperature nephograms of a heater with the same heating power. It can be found that the electric heating rod surface temperature gradually rises along the direction of air flow. With the increase of the diameter of the electric heating rod, the maximum temperature of the heating rod surface first increases and then decreases. The maximum surface temperature of H110-20 is 1.40 and 4.98% higher than those of H110-16 and H110-24, respectively.

**Figure 8:**
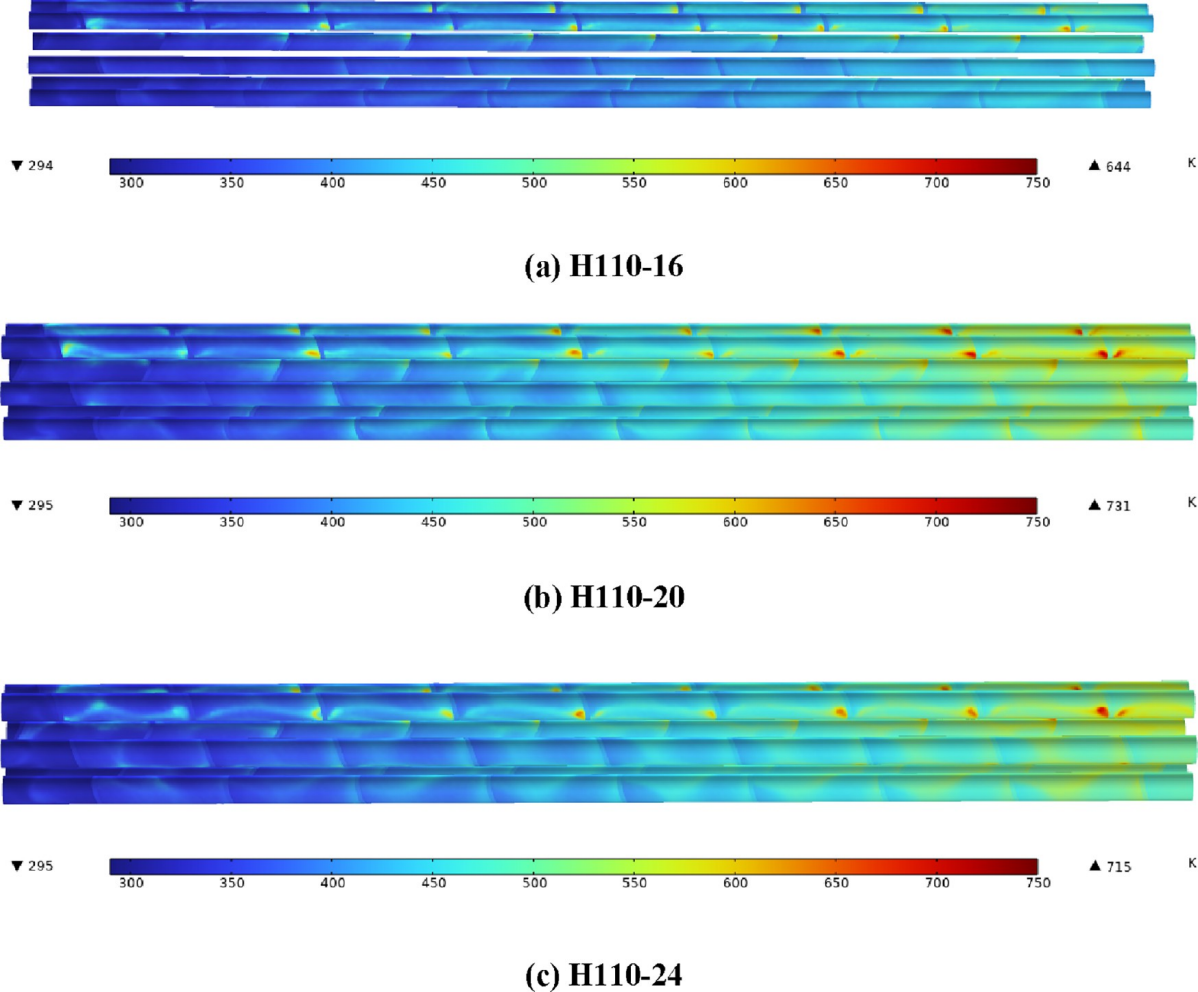
(a–c) Electric heating rod surface temperature nephograms.

Figure 8 shows the surface temperature nephograms of the heater with the same heat flux. The variation trend is consistent with that of Figure 7. Compared with H110-16, the maximum surface temperature of the electric heating rod of H110-20 and H110-24 is increased by 13.51 and 11.02%, while the heating power is increased by 25.0 and 50.0%, respectively. Compared with H110-20, the increase of shell-side heat transfer capacity of H110-24 is greater than that of heating power, so the increase of surface temperature of H110-24 is lower. The increase of the surface temperature of the electric heating rod is much smaller than that of the heating power, which shows that increasing the diameter of the heating rod can effectively reduce the surface temperature of the heating rod with high heating power.

**Figure 9:**
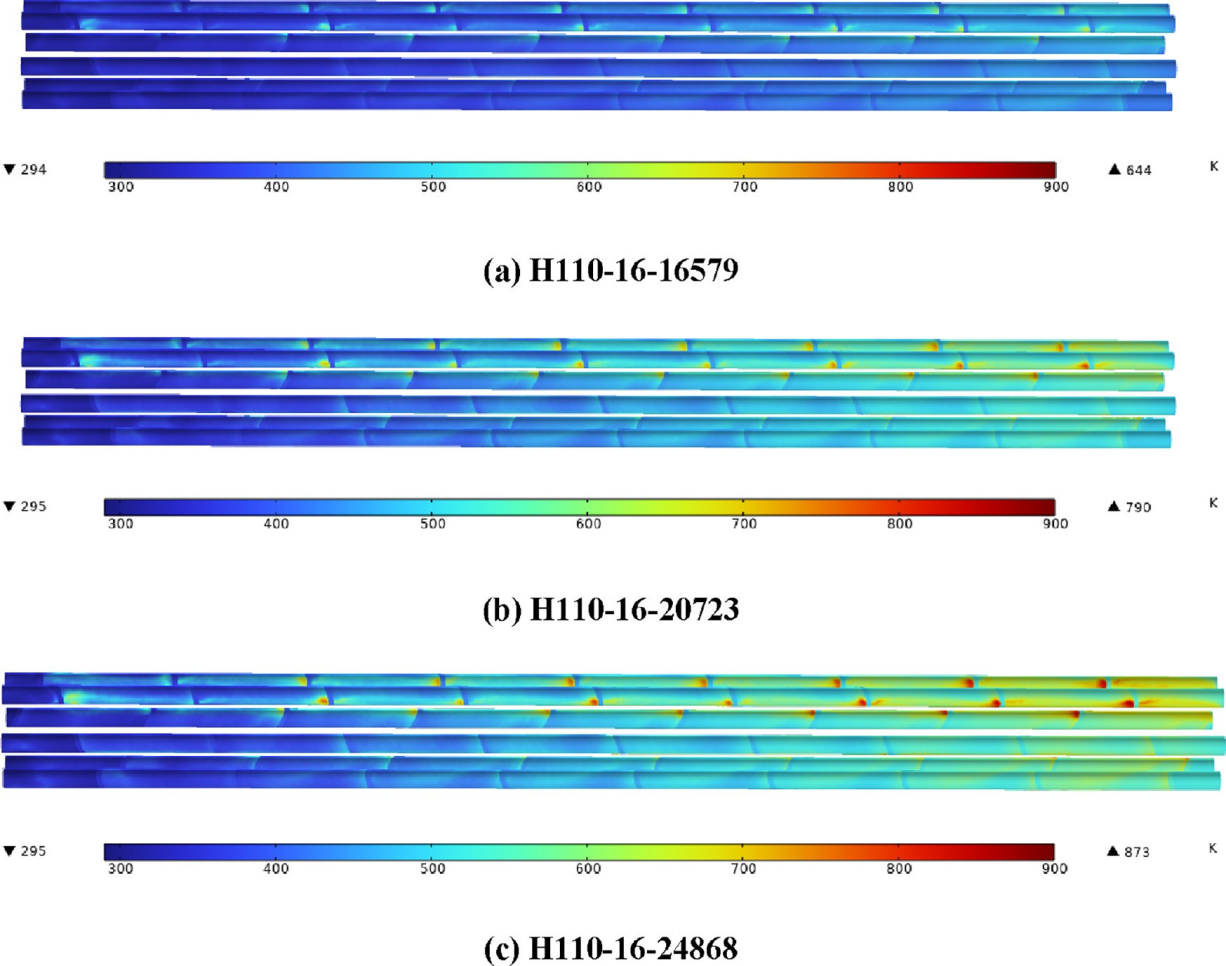
(a–c) Surface temperature nephograms of H110 with different heat flux.

To further clarify the influence of heat flux on the heating rod surface temperature, three different heat flux values are set for H110-16. In Figure 9, the heating power of H110-16-20723 and H110-16-24868 is the same as that of H110-20 and H110-24 in Figure 8, respectively. The maximum surface temperature of the electric heating rod of H110-16-20723 and H110-16-24868 is 22.67 and 35.56% higher than that of H110-16-16579, respectively, which is higher than that of H110-20 and H110-24 in Figure 8. Figures 7–9 show that increasing the diameter of the electric heating rod can not only effectively reduce the surface temperature of the electric heating rod but also reduce the growth range of the surface temperature of the electric heating rod during high-power heating.

### 3.3 Average Temperatures of the Electric Heating Rod

**Figure 10:**
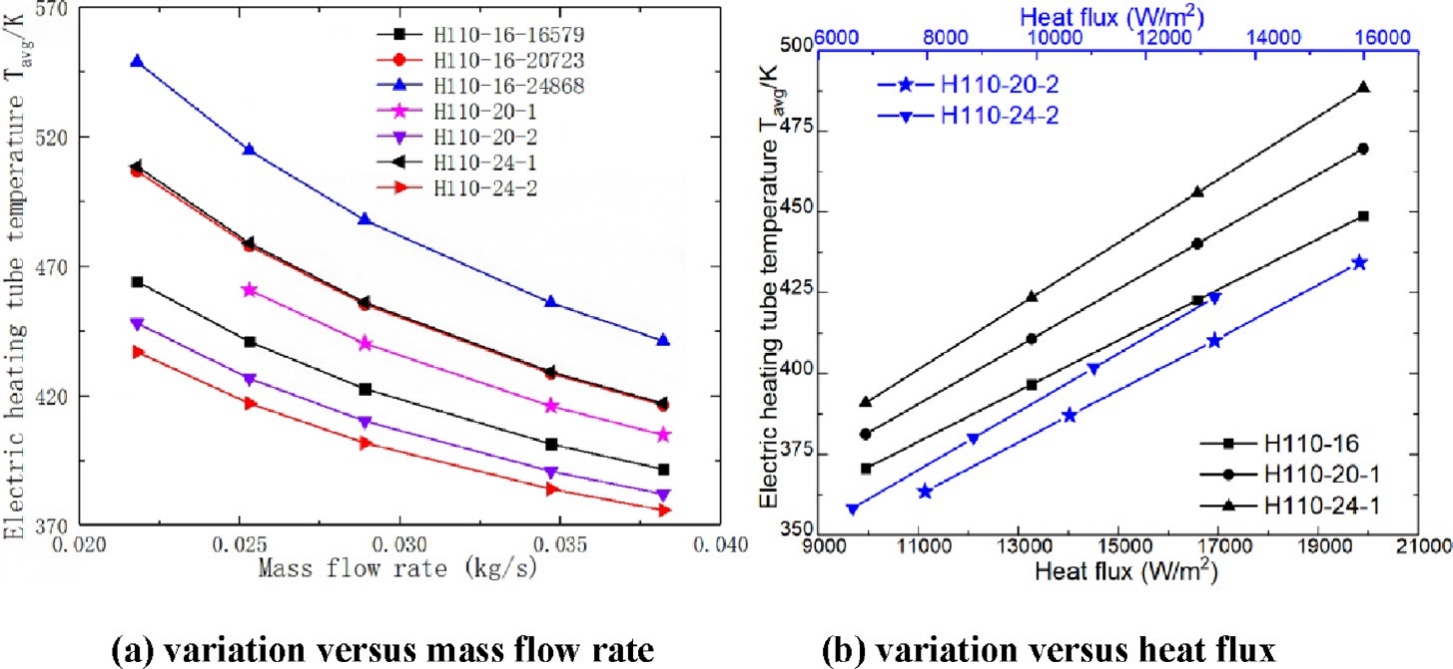
Average temperature variation trend of electric heating rods (a,b).

**Figure 11:**
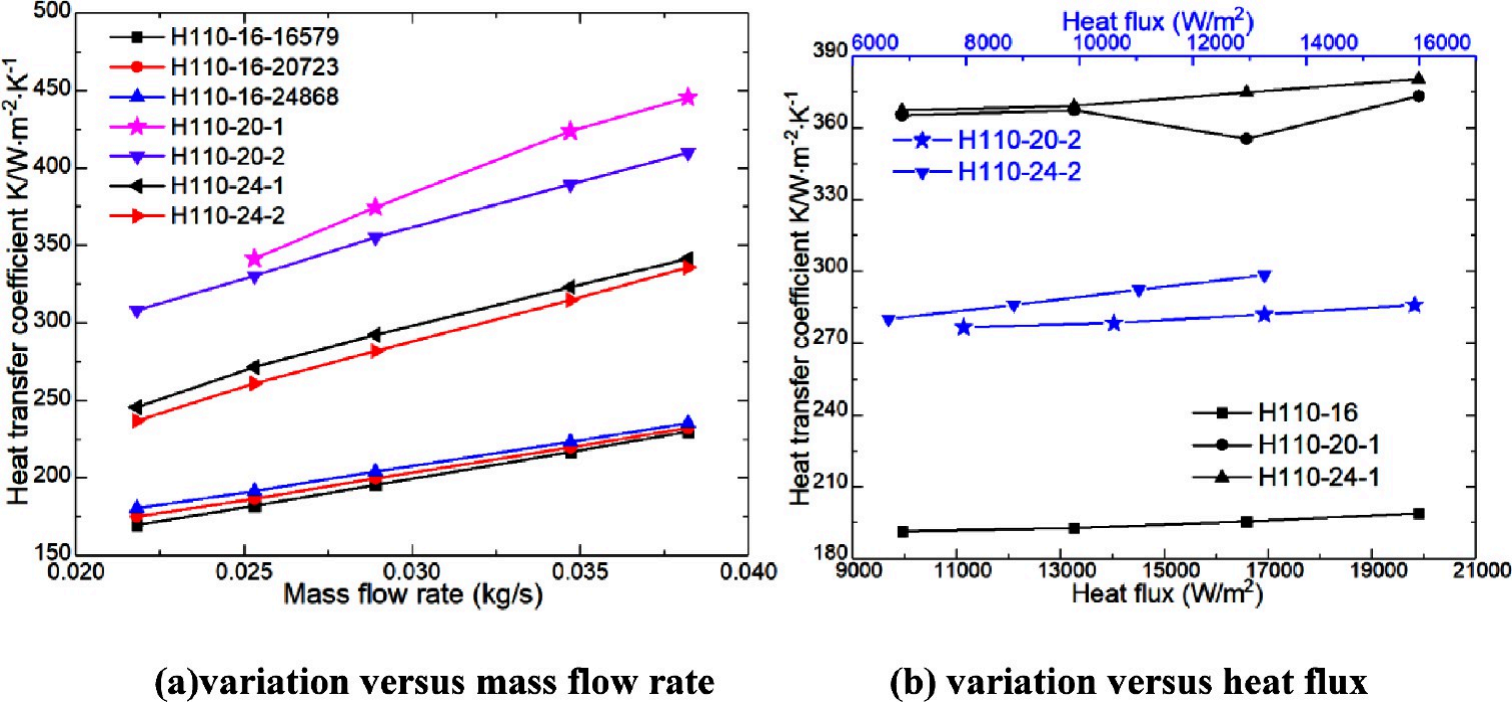
Heat transfer coefficient of the heater (a,b).

Figure 10 presents the variation trend of the average temperature of an electric heating rod under different heating parameters. We found that the average temperature of an electric heating rod with a mass flow rate and heat flux shows an opposite trend. In addition, as the mass flow rate increases, the air velocity on the shell side also increases, thereby enhancing convective heat transfer between the air and the surface of the electric heating rod. Consequently, with an increase in the mass flow rate, there is a gradual decrease in average temperature of the electric heating rod. On the other hand, with an increase in heat flux, although more power is absorbed by the air on the shell side, it leads to a rapid increase in average surface temperature of the electric rod. At equivalent heat flux levels, H110-24-16579 and H110-20-16579 exhibit 6.50–9.58% and 3.40–4.52% higher surface temperatures compared to H110-16-16579, respectively. The heating power of H110-24-11025 and H110-20-13263 is identical to that of H110-16-16579, and they are 4.03–5.86% and 2.46–3.21% lower than that of H110-16-16579, respectively. The heating power of H110-16-20723 and H110-16-24868 is the same as that of H110-24-16579 and H110-20-16579, respectively. The average surface temperature of H110-16-20723 and H110-16-24868 is 2.84–3.71% and 5.78–7.93% higher than that of H110-24-16579 and H110-20-16579, respectively. The average surface temperature of H110-16-20723 and H110-16-24868 is 6.33–9.17% and 12.6–18.27% higher than that of H110-16-16579, respectively. As shown in Figure 11, the variation trend of the heating rod surface temperature under different heating rod diameters and heating parameters indicates that increasing the diameter of the heating rod is an effective way to reduce the heating rod surface temperature during high heating power.

### 3.4 Heat Transfer Coefficients

The variation trend of the heat transfer coefficient with the heating parameters is illustrated in Figure 11. It can be observed from Figure 11 that the heat transfer coefficient exhibits a gradual increase with an increase in the mass flow rate while demonstrating a slight upward trend with an increase in heating power. In addition, as the mass flow rate increases, the air velocity on the shell side also increases, thereby enhancing convective heat transfer between the air and the electric heating rod surface. The increase of enhanced heat transfer capacity is slightly higher than that of heat flux, so the heat transfer coefficient shows a slight upward trend. In Figure 11a, the higher the heat flux, the higher the heat transfer coefficient. When the diameter of the heating rod is 20 mm, the heat transfer coefficient is the highest and the increase is the largest. At the same heat flux, the heat transfer coefficients of H110-24 and H110-20 are 44.82–48.49% and 87.52–95.48% higher than those of H110-16, respectively. With the same heating power, the heat transfer coefficients of H110-24 and H110-20 are 39.69–46.06% and 78.25–81.74% higher than those of H110-16, respectively. The heat transfer coefficients of H110-24-16579 and H110-20-16579 are 36.08–45.04% and 82.99–92.84% higher than those of H110-16-20723 and H110-16-24868. The heat transfer coefficients of H110-16-20723 and H110-16-24868 are 1.05–3.28% and 2.38–6.42% higher than those of H110-16-16579, respectively. This shows that the heat transfer coefficient of the heating rod can be effectively improved by increasing the diameter of the electric rod.

### 3.5 Pressure Drops

**Figure 12:**
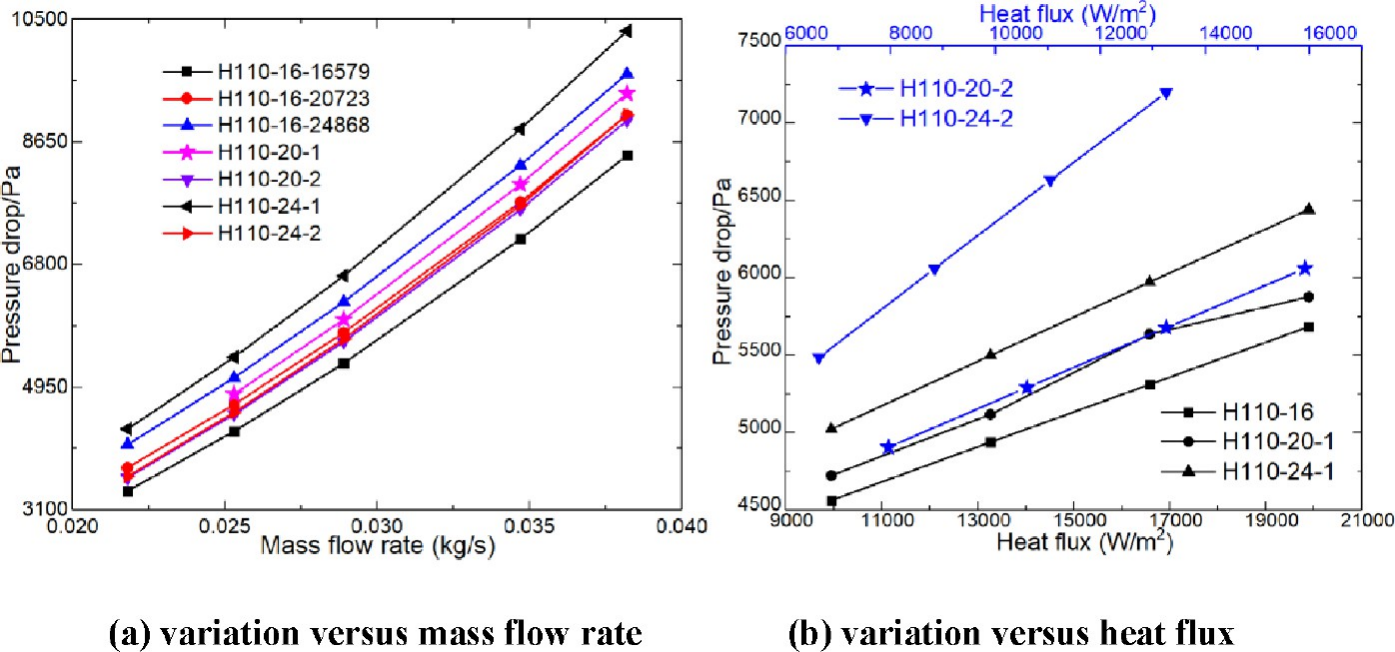
Pressure drop of the heater (a,b).

Figure 12 shows the variation trend of the pressure drop with heating parameters. As shown in Figure 12, the pressure drop increases with the increase of heating parameters, and the influence of the mass flow rate on the pressure drop is greater than that of heating power. With the increase of the mass flow rate, the growth rate of the pressure drop gradually increases, while with the increase of heating power, the growth rate of the pressure drop is basically unchanged. The pressure drop of the heater shell includes the local pressure difference resistance and the friction resistance along the shell side. The local pressure difference resistance is mainly generated at the leeward side of the electric heating rod, and the friction resistance along the shell side is generated at the contact surface between the air and the heater. As shown in Figure 12, the greater the heat flux, the greater the pressure drop. This is because the heating power increases, the air temperature increases, the viscosity increases, and the local pressure difference resistance and friction resistance along the way increase simultaneously. The larger the diameter of the electric heating rod, the larger the pressure drop and the higher the change rate of the pressure drop. Under the same heat flux, the pressure drops of H110-24 and H110-20 are 27.56–22.28% and 11.08–13.13% higher than that of H110-16, respectively. With the same heating power, the pressure drops of H110-24 and H110-20 are 6.44–7.26% and 5.92–6.30% higher than that of H110-16, respectively. The pressure drops of H110-24-16579 and H110-20-16579 are 6.72–5.80% and 3.32–3.52% higher than those of H110-16-20723 and H110-16-24868, respectively. The pressure drops of H110-16-20723 and H110-16-24868 are 7.3–10.33% and 14.58–20.56% higher than those of H110-16-16579, respectively.

### 3.6 Comprehensive Performances

**Figure 13:**
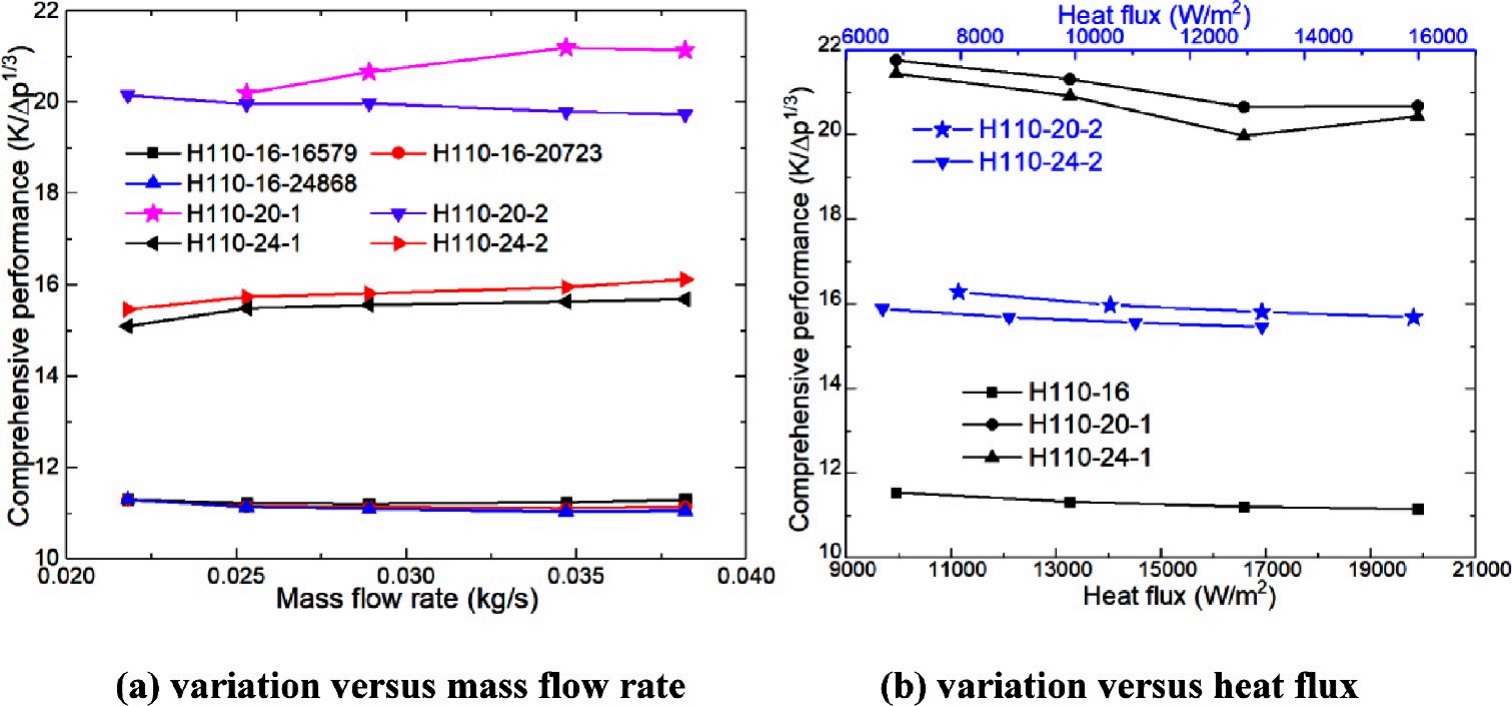
Traditional comprehensive performance of the heater (a,b).

Figure 13 shows the variation trend of the traditional comprehensive performance with heating parameters. In Figure 13a,b, the traditional comprehensive performance increases slightly with the increase of the mass flow rate but shows a downward trend with the increase of heat flux. Under the same conditions, the traditional comprehensive performance of H110-20 is the highest followed by H110-24, and H110-16 is the lowest. Under the same heat flux, the traditional comprehensive performance of H110-24 and H110-20 is 33.53–38.86% and 78.18–87.06% higher than that of H110-16, respectively. With the same heating power, the traditional comprehensive performance of H110-24 and H110-20 is 36.81–42.68% and 74.65–88.48% higher than that of H110-16, respectively. The traditional comprehensive performance of H110-24-16579 and H110-20-16579 is 33.54–41.92% and 81.01–89.53% higher than that of H110-16-20723 and H110-16-24868, respectively. The traditional comprehensive properties of H110-16-16579 are 0.05–1.32% and 0.01–2.21% higher than those of H110-16-20723 and H110-16-24868, respectively.

**Figure 14:**
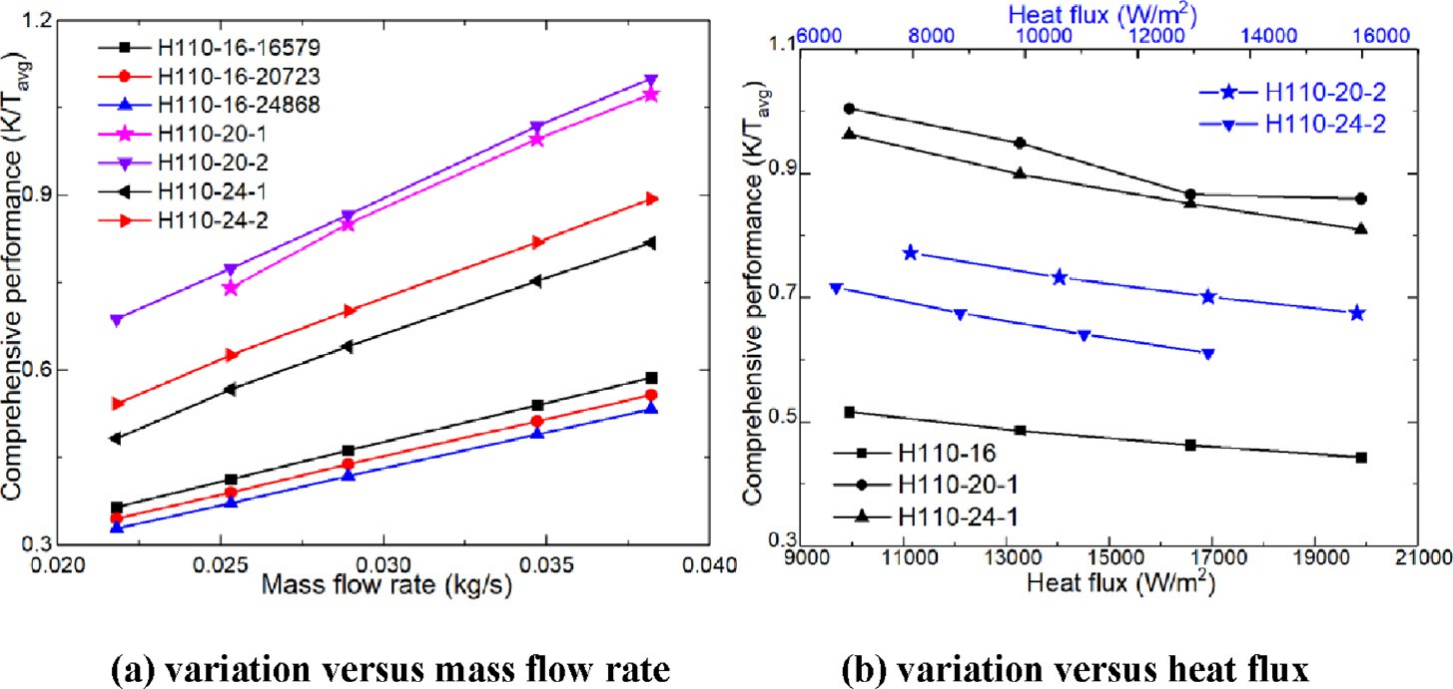
New comprehensive performance of the heater (a,b).

Figure 14 illustrates the trend of the new comprehensive performance with heating parameters. As shown in Figure 14a,b, the new comprehensive performance exhibits a clear increasing trend with the rise of the mass flow rate, while it demonstrates a noticeable decreasing trend with the increase of heat flux. The variation trend of different heaters with different heating parameters is basically consistent with the trend in Figure 13. Under the same heat flux, the new comprehensive performance of H110-24 and H110-20 is 32.16–39.43% and 82.66–86.40% higher than that of H110-16, respectively. With the same heating power, the new comprehensive performance of H110-24 and H110-20 is 48.38–52.34% and 87.29–95.19% higher than that of H110-16, respectively. The new comprehensive performance of H110-24-16579 and H110-20-16579 is 46.87–53.68% and 89.77–94.40% higher than that of H110-16-20723 and H110-16-24868, respectively. The new comprehensive performance of H110-16-16579 is 5.23–5.78% and 10.04–11.03% higher than that of H110-16-20723 and H110-16-24868, respectively. The rules in Figures 13 and 14 show that increasing the heating power can improve the comprehensive performance of the heater, but the most effective way is to increase the diameter of the heating rod.

## 4 Conclusions

In this work, the influence of the electric heating rod diameter on heater performances is studied by numerical simulations, and the flow and temperature characteristics of the heater are analyzed in detail. Finally, the optimum diameter of the electric heating rod is obtained through the comprehensive evaluation of the heater. Based on the results, we found the following conclusions.

1. Increasing the diameter of the heating rod helps to increase the minimum and maximum velocity of the shell-side air, where the maximum velocity of H110-24 is 16.34 m/s, which is 1.25 and 1.13 times those of H110-16 and H110-20, respectively.
2. The location of the local high temperature zone coincides with the area with low air flow velocity, and increasing the diameter of the heating rod is an effective way to reduce the heating rod surface temperature during high heating power.
3. Increasing the heating power can improve the comprehensive performance of the heater, but the most effective way is to increase the diameter of the heating rod. With the same heating power, the new comprehensive performance of H110-24 and H110-20 is 48.38–52.34% and 87.29–95.19% higher than that of H110-16, respectively, and the electric heating rod with the diameter of 20 mm has the best performance.

## Nomenclature

A: area, m^2^
*h*: heat transfer coefficient, W/(m^2^·K)
*K*: comprehensive performance
*k*: turbulent fluctuation kinetic energy, m^2^/s^2^
*P*: heating power, W
*p*: pressure, Pa
*S*: source term
*U*: velocity vector

## Greek Symbols

ρ: density, kg/m^3^
Γ: generalized diffusion coefficient
ε: turbulent kinetic energy dissipation rate, m^2^/s^3^
Δ*p*: pressure drop, Pa
Δ*T*: log mean temperature difference, K
Φ: variables in generalized equations

## Subscripts

avg: average
hr: heating rod
in: inlet
max: maximum
min: minimum
out: outlet
ss: shell-side
trad: traditional
new: new
